# Equine Suture Exostosis: A Review of Cases from a Multicenter Retrospective Study

**DOI:** 10.3390/vetsci9070365

**Published:** 2022-07-17

**Authors:** Denis Verwilghen, Jack Easley, Timo Zwick, Maggy Uhlhorn, Sigrid Grulke, Hubert Simhofer, Neil Townsend, Oliver Liyou, Fabrice Bodeus, Davide Danilo Zani, Lieven Vlaminck, Chris Pearce, Carsten Staszyk, Astrid Bienert-Zeit

**Affiliations:** 1 Sydney School of Veterinary Science, University of Sydney, Camperdown, NSW 2050, Australia; 2 Goulburn Valley Equine Hospital, School of Veterinary Sciences, University of Melbourne, Melbourne, VIC 3010, Australia; 3 Easley Equine Dentistry, Shelbyville, KY 40065, USA; easleydvm@aol.com; 4 Equine Clinic Gessertshausen, 86459 Gessertshausen, Germany; t.zwick@tierklinik-gessertshausen.de; 5 Imaging Department, Swedish University of Agricultural Sciences, 75105 Uppsala, Sweden; margareta.uhlhorn@uds.slu.se; 6 Equine Clinic, Faculty of Veterinary Medicine, University of Liege, 4000 Liege, Belgium; sgrulke@ulg.ac.be; 7 Equine-Vets, 2371 Hinterbruhl, Austria; info@equine-vets.at; 8 Three Counties Equine Hospital, Stratford Bridge, Ripple, Tewkesbury, Gloucestershire WR13 6NW, UK; nt8261@icloud.com; 9 Equine Veterinary Dental Services, Grafton, NSW 2460, Australia; oliver@evds.net.au; 10 Centre Vétérinaire des Templiers, 6460 Chimay, Belgium; bodeus.flamme@skynet.be; 11 Veterinary Teaching Hospital, Department of Veterinary Medicine and Animal Sciences (DIVAS), Università degli Studi di Milano, 26900 Lodi, Italy; davide.zani@unimi.it; 12 Department of Surgery and Anaesthesia, Faculty of Veterinary Medicine, University of Gent, 9820 Merelbeke, Belgium; lieven.vlaminck@ugent.be; 13 Equine Dental Clinic, Dorset WR13 6NW, UK; pearce.cj@gmail.com; 14 Institute of Veterinary-Anatomy, -Histology and -Embryology, Faculty of Veterinary Medicine, Justus-Liebig-University Giessen, Frankfurter Str. 98, 35390 Giessen, Germany; carsten.staszyk@vetmed.uni-giessen.de; 15 Clinic for Horses, University of Veterinary Medicine Hannover, 30559 Hannover, Germany; astrid.bienert@tiho-hannover.de

**Keywords:** horse, bone sutures, facial, sequestra, infection, maxillofacial, dentistry

## Abstract

**Simple summary:**

Suture exostosis is a condition affecting the horse’s head. The connections between the bone plates that form the horse’s face have been shown to react to insult. Horses will then develop a swelling along the face that may be painful. Little is known about this condition and the present research project aimed to investigate horses presented to equine clinics with symptoms of the disorder. It was revealed that the condition can form following trauma, underlying sinus disease, following a surgery or without any apparent cause. Various treatment options to resolve the condition have been reported and the outcomes of those are described in the paper. Most consistently the proper diagnosis and identification and removal of potential bone sequestra are crucial for a timely resolution.

**Abstract:**

Suture exostosis is an intriguing and not uncommon pathology that has to be included in the differential diagnosis for horses with swelling of the head. Although several singular case reports have been published, no large case series is available. The aim of this study is to report a multicentric retrospective collection of suture exostosis cases. Data concerning horses with suture exostosis in the facial region were collected retrospectively. Information regarding breed, age, gender, history, imaging findings, initiated treatment, response to treatment and follow up was recorded. One hundred and five cases of various breeds were reported. Analysis revealed the cases could be grouped into four entities: 45 developed following sino-nasal surgery, 23 following trauma, seven with underlying sinus pathology and 25 idiopathic. Treatment consisted of sequestra removal, plate fixation, antimicrobial and anti-inflammatory drugs or no treatment. Whereas initial localized pain fades within few days or weeks, resolution or reduction of the swelling was obtained in most cases after 3 months to 1.5 years. The etiopathogenesis of suture exostosis seems to consist of different entities. Identification of an underlying cause, particularly the presence of a bone sequester and infection is important to speed up resolution and before concluding an idiopathic case. When performing sinusotomies, it is important to provide as little trauma as possible to the surgical site in order to prevent suture exostosis as a complication.

## 1. Introduction

Facial suture exostosis, suture periostitis, suture separation, or suturitis is an intriguing and not so uncommon condition that should be included in the differential diagnosis of equine facial swelling [1]. Horses affected with suture exostosis are reported to gradually develop uni- or bilateral, firm, non-painful swelling in the frontal region of the head. The exact location of the swelling is dependent on the facial suture or sutures affected [2] The problem is usually diagnosed from clinical signs and radiographs. Radiographs often reveal a radiolucent suture line surrounded by an area of increased opacity and callus formation [3]. Because of the complex three-dimensional anatomy of the head and the difficulties in interpreting radiographs due to the overlapping of several bony structures, CT offers a better image of the problem. CT usually shows irregular new periosteal reaction and proliferation with a cloudy appearance along involved suture lines. New bone formation is often bilateral, symmetric and extends to the orbital region [4].

The condition is thought to be self-limiting and spontaneously resolves over time without treatment. Common complications are chronic epiphora if the naso-lacrimal or lacrimo-maxillary suture is involved and chronic draining tracts if a bone sequester is present. In some cases, persistent instability is mentioned to result in a progressive increase in the size of swellings. In chronic complicated cases, surgical treatment with local debridement of bone sequestra or even stabilization of the sinus sutures with bone plates has been used to resolve the problem [2,5].

Several singular case reports of horses affected with the condition have been published [3,5,6,7,8], but no larger case reviews with follow-up are available. The aim of this multicenter retrospective study is to collect information on clinical cases of facial suture exostosis.

## 2. Materials and Methods

A multicentric study was established in which data concerning horses reported with suture exostosis in the facial region were collected retrospectively. Any horses diagnosed with facial suture exostosis presented at one of the authors’ clinics for which clinical information could be retrieved were included in the study. Information regarding breed, age, gender, history of trauma, history of surgery, history of sinus pathology, imaging findings, initiated treatment, response to treatment and follow up data were collected. 

## 3. Results

### 3.1. Case Characteristics

One hundred and five cases have been cataloged, originating from Europe, North America, and Australia. The breed, gender and age distribution are reported in Table 1. Season of appearance and housing characteristics are described in Table 2. No particular housing or season predisposition seems to be reported. From history the cases could be grouped into four categories as described in Table 3. The onset of swelling varied from a few days to 44 weeks of duration (mean 5.67 weeks–median 4 weeks). Horses identified to have had trauma witnessed have reported intensity to vary from “hit the head when backed out of the trailer” to open impression fractures. 

**Figure 1:**
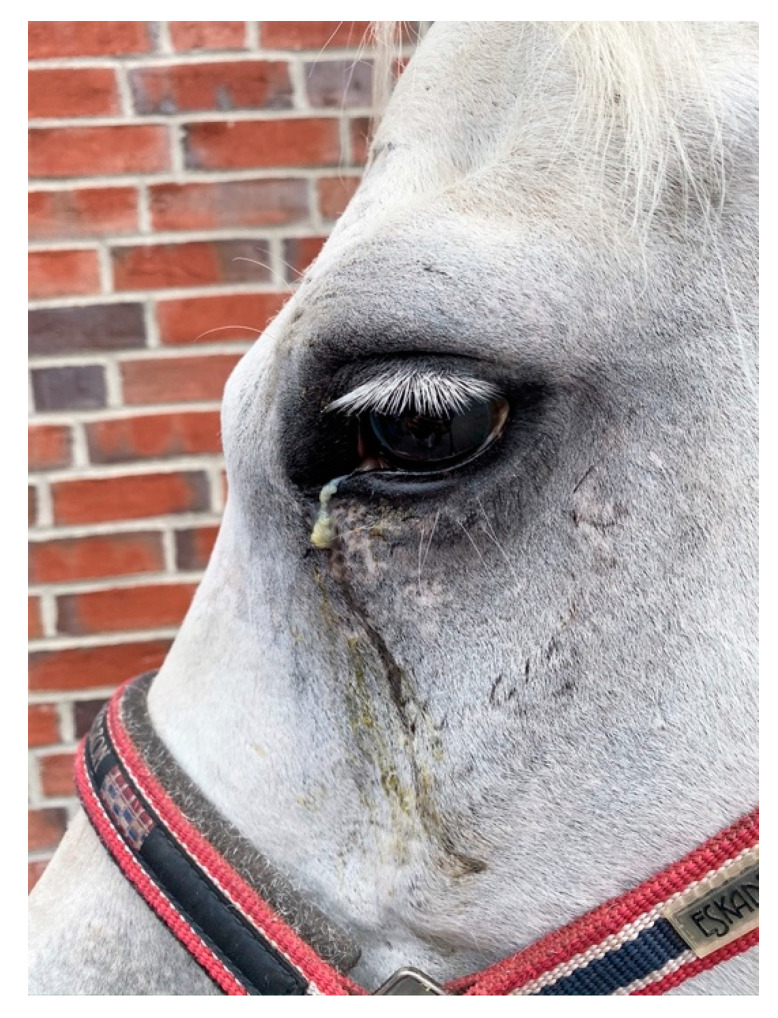
Picture of a horse with moderate, purulent epiphora secondary to idiopathic suture exostosis.

**Figure 2:**
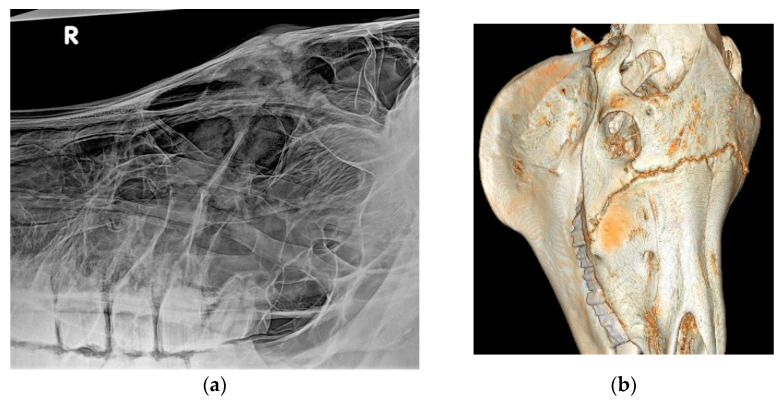
(**a**,**b**): X-ray (**a**) and CT (**b**) reconstruction of a horse with severe suture exostosis secondary to a right sided dental sinusitis. Sinus disease was caused by chronic diastema formation between 108/109 and 109/110 and severe alveolitis.

A total of 39 horses showed epiphora (Figure 1) and 35 had nasal discharge. The distribution of nasal discharge and epiphora per history category are described in Table 4. Further to these more common findings related to suture exostosis, one horse presented with a severe overjet and one with headshaking, both of which had an idiopathic origin of the swelling. One horse in the sinus disease group had severe dental malocclusions leading to secondary sinusitis (Figure 2a,b). No other details on dental occlusion status were available for the reviewed cases. Horses examined in an acute phase of presentation, particularly following trauma or post-surgery, presented with pain at local palpation of the swollen suture lines. 

**Figure 3:**
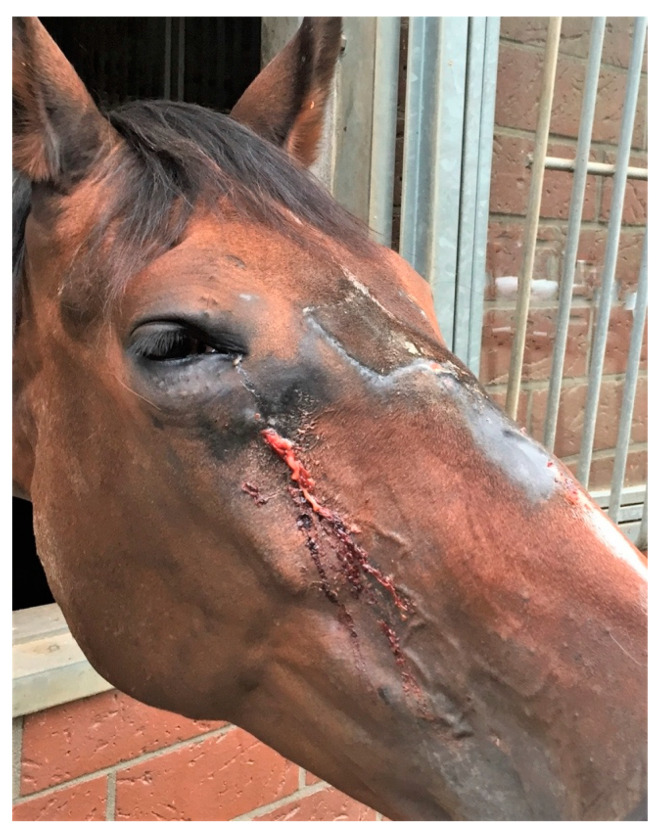
Mare after bone flap surgery with secondary suture exostosis leading to bacterial infection and abscess formation on the nasal bridge and under the right eye.

From the 48 cases developing suture exostosis following surgery, the surgical approach to the sinus is described in Table 5. From the 25 flap surgeries (Figure 3), 11 were performed with an oscillating saw, two with chisel and in 12 the method was not reported.

From the eight cases in which the swelling coincided with the presence of an underlying sinus disease, in six a soft tissue mass or cyst was identified, in one a secondary dental sinusitis was present and in one a primary sinusitis was identified (bilateral sinus fluid filling). 

### 3.2. Imaging Findings

**Figure 4:**
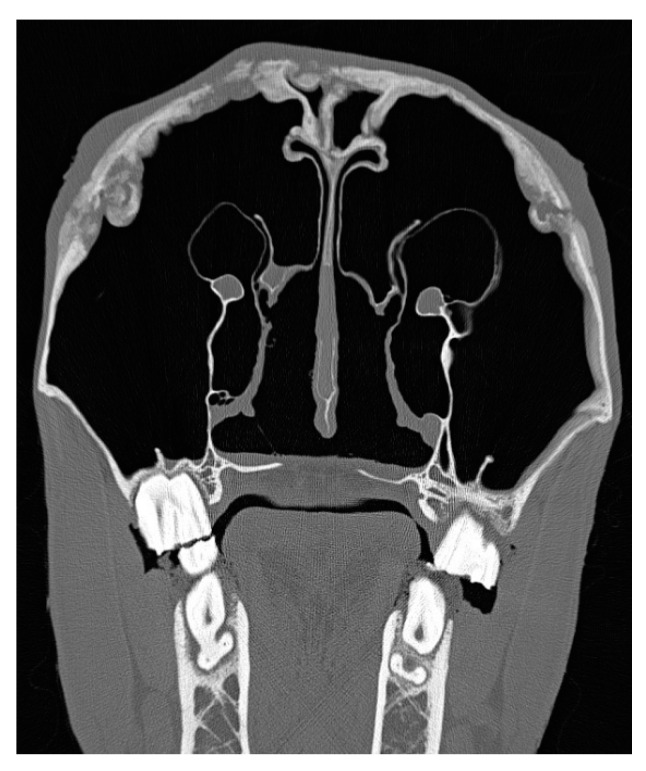
Transversal CT scan of a horse with bilateral idiopathic suture exostosis involving both nasolacrimal ducts. Reactions of sinus mucosa and skin are only mild.

**Figure 5:**
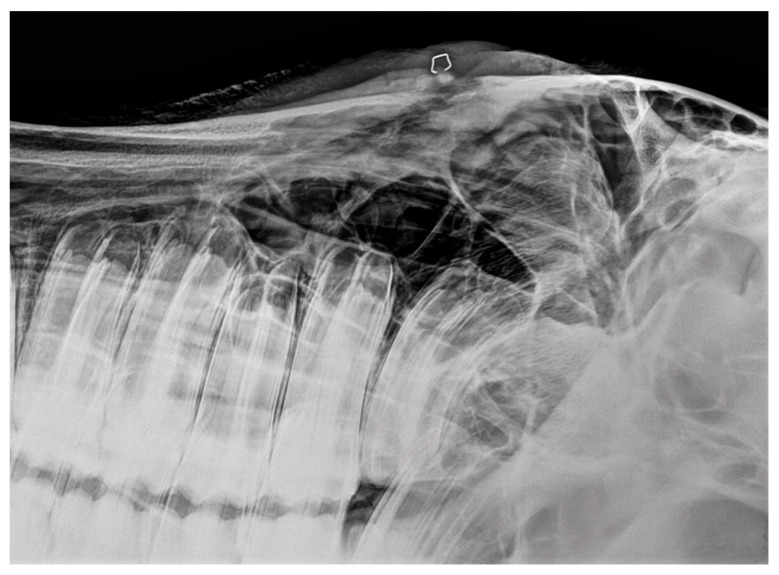
X-ray of a young horse with severe suture exostosis with sequester formation (metallic marker) and moderate soft tissue swelling.

**Figure 6:**
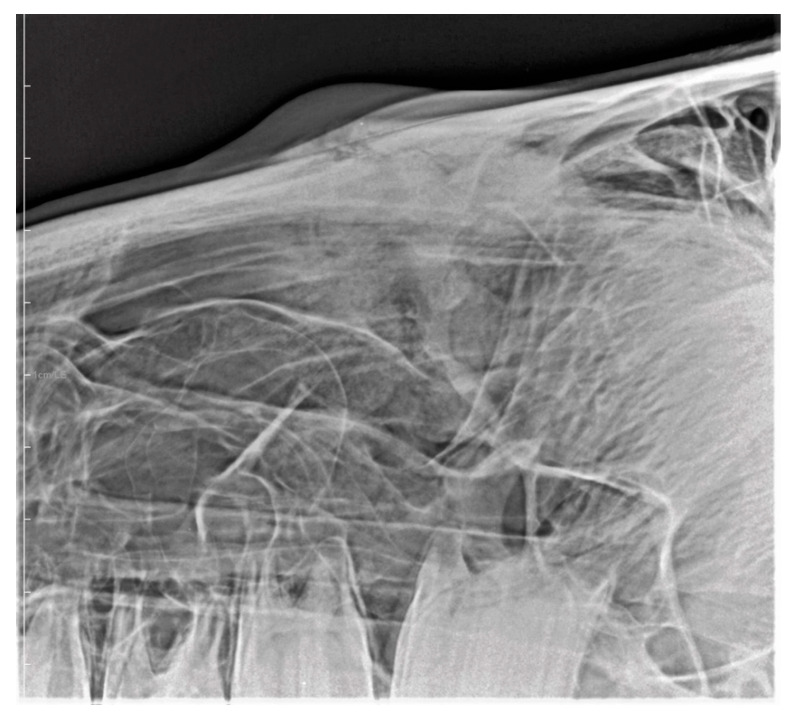
X-ray of a horse with mild to moderate, but painful suture exostosis with sequester formation.

**Figure 7:**
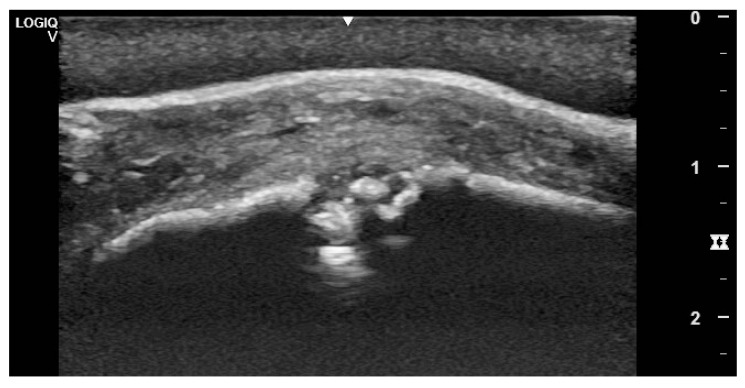
Ultrasonographic appearance of suture exostosis with central sequester formation. Note the thickened periosteum, subcutaneous tissue and skin.

Eight cases underwent computed tomography of the head (Figure 4); four did not have any imaging performed and for the remainder of the cases radiographs were available (Figure 5). The most common imaging description was a smoothly outlined periosteal and endosteal proliferation in the area of the suture lines with mild to moderate soft tissue swelling surrounding the periosteal reaction. Various degrees of suture separation were reported. In 28 cases, one or more bone sequestra were identified on imaging (Figure 6) (presence of sequestra per history described in Table 6). In two, an ultrasound was needed to confirm the presence and localization of the sequestra (Figure 7).

### 3.3. Treatments

**Figure 8:**
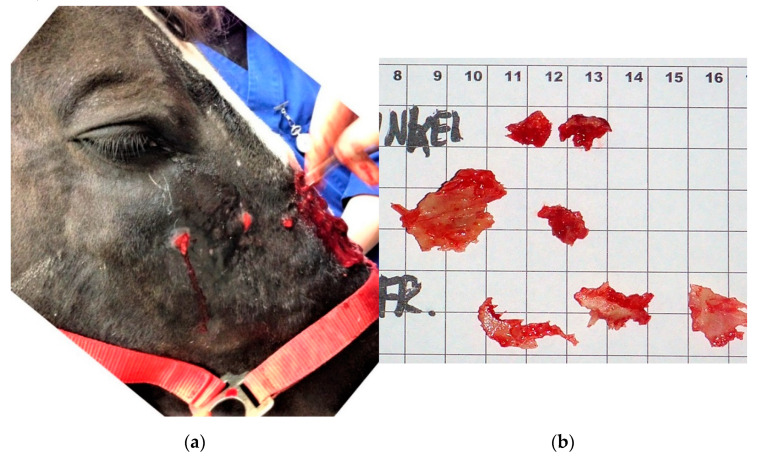
(**a**,**b**): Surgical debridement and sequestrectomy in a horse with moderate suture exostosis forming purulent abscesses after bone flap surgery.

**Figure 9:**
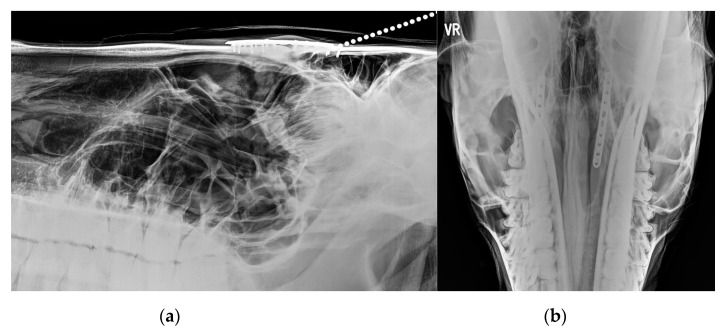
(**a**,**b**): LL (**a**) and DV (**b**) X-ray of one of the horses with internal fixation of the suture lines after surgery. Note two 10-hole plates positioned 2.5 cm on the left and right of the midline.

Combinations of treatments were reported. Surgical debridement and sequestrectomy (Figure 8a,b) were performed in 19 cases in which infection and sequestration was identified post sinusotomy. Six of 23 trauma cases underwent sequestrectomy and all were treated with local and systemic NSAIDs. Horses with underlying sinus diseases were treated by mass/cyst removal in six, sinus flush in one, and one was euthanized on the owner’s request due to the severity of dental disease and associated sinusitis. Cases with idiopathic appearance of the swelling received various treatments including no treatment, corticosteroids, NSAIDs and topical anti-inflammatories. Most horses were advised to stall rest with hand walking, and some had diet changes advised with the omission of carrots and other hard food. Two cases underwent a fixation of the suture lines, in one using a double plating with 10 hole 2.7 mm DCP plates, the other using an LC-DCP plate (Figure 9a,b). 

### 3.4. Follow up—Outcomes

One case was euthanized after initial consult. The follow up data on 88/104 cases are described in Table 7. Persistence of epiphora or nasal discharge was not reported in any of the cases, where follow up information is available. 

## 4. Discussion

The clinical expression of the condition suture periostitis, exostosis, separation or suturitis is likely an expression of different etiopathogenic entities [2] which is confirmed in the review of the present cases where the condition is reported in four distinct categories: post trauma, post sinonasal surgery, in conjunction with a sinus disease, and without any observed or reported reason (idiopathic). Any type of head trauma or “stress” to the suture lines, leading to instability of the cranial sutures and/or any inflammatory reaction in the region has the potential to lead to the development of the condition. Pure traumatic insult to the head, leading to facial bone fractures and instability will eventually lead to callus formation as a natural response to fracture stabilization. Equally, surgical insult created during sinusotomy by either bone flap or trephination, or infection of the suture lines following a sinusotomy can result in instability and perio/endostal reaction leading to bone proliferation and a similar clinical expression. 

The morphogenesis of cranial and facial bones is a complex and lengthy developmental process initiated during early embryogenesis and reported to be completed during adulthood. During the fetal period, the bones of the calvaria (top of the skull) and face are formed from intramembranous ossification sites within the mesenchyme covering the brain [9]. In the facial area, these bones remain separated by connective tissue that will later develop into immovable “joints” called sutures.

From an anatomical point of view this construction represents a non-synovial joint, i.e., a syndesmosis. During development the bones enlarge, and the connective tissue areas become reduced. Finally, bones approach each other and form borders featuring interdigitating lines. This bony formation is referred to as a cranial suture. Anatomical terms indicate exactly which facial bones are connected by a suture, e.g., Sutura internasalis, Sutura lacrimomaxillaris etc. It shall be emphasized that connective tissue remains present within a suture for a long time, although it is rarely visible macroscopically. Therefore, cranial suture should be regarded as almost immovable “non- synovial joints” in terms of syndesmoses.

Most sutures of the face remain open for large periods of time and in young animals they take the appearance of irregular lines which will eventually disappear by osseous fusion in the aged animal [9]. The exact timing is well described in humans, since it is often used in paleontological research, but we lack comprehensive data in equids. Nevertheless, in a study investigating normal suture lines in horses (*n* = 52) aged 2 to 30 years it has been shown that the equine cranial sutures feature the same histological characteristics of a syndesmosis as described in man and other non-equine species [10]; the content of which can be described as a fibrous joint filled with connective tissue rich in collagen that is highly cellular and vascularized [10].

Regarding the clinically most relevant sutures of the face, it was shown that only the frontomaxillary suture expresses complete bony fusion in horses older than 20 years. The lacrimomaxillary and zygomaticomaxillary sutures remained at least partly filled with connective tissue even in some individuals of up to 30 years of age. The internasal suture seems to remain unfused in the majority of the investigated horses [10]. Although a completed fusion of the sutures has been assessed by high resolution computed tomography, confirmed only by a number of selective histological investigations, it remains uncertain whether in this study [10] some remnants of connective tissue may have been missed in sutures classified as completely fused.

**Figure 10:**
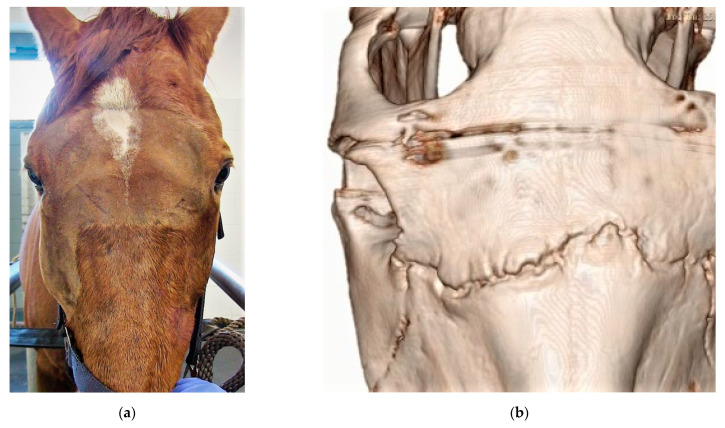
(**a**,**b**): Clinical situation (**a**) and CT (**b**) reconstruction of a horses with bilateral, nearly symmetric suture exostosis, starting at one side, advancing along the suture lines to the contralateral side.

From the latter findings [10] it seems the suture lines in fact represent a patent way for inflammation to travel across as can be seen in the clinical expression of the condition. It is therefore not unusual for the insult, infection or sequester to be located on one side of the face yet the reaction of the suture line to be present bilaterally (Figure 10a,b). The suture line seems to be able to act as a highway for infection and inflammation.

### 4.1. Trauma Cases

The degree of trauma appears to be independent of the manifestation of the suture exostosis. The latter can be nicely illustrated from the 23 cases in which trauma was identified. Most particularly, one case was seen to hit its head when backing out of the trailer with an absence of open wounds or identifiable facial depressions. In the following weeks the swelling appeared, and radiographs showed sclerosis and bone proliferation over the frontonasal suture. This is in contrast to traumatic cases in which clear open trauma occurred with presence of sequestra and the development of infection before the appearance of the swelling. Tremaine and Dixon [11] in their review of sinusitis cases reported five cases of traumatic induced suture exostosis from 15 cases presented with secondary sinusitis with facial bone fractures.

In the cases where a traumatic insult was identified, it is expected that surgical stabilization of the suture line will overcome the instability and allow for faster and more complete healing of the suture/fracture site. Although applied in two idiopathic cases in this series, it was not used for traumatic cases.

### 4.2. Post Sinusotomy Cases

In nearly half of the reported cases (*n* = 48) the facial swelling appeared as a complication of sinusotomy. This is consistent with reviews on sinusitis management, which often mention the occurrence of suture exostosis in the enumeration of surgical complications [11,12,13]. Whereas the follow up of affected cases following sinusotomy is reported to show good resolution with a conservative approach (Tremaine and Dixon 2001), the current case series reveals that the identification and removal of sequestra and the treatment of any associated infection appears to be crucial in bringing resolution of the suture periostitis in the post sinusotomy cases. Eighteen cases had one or more sequestra identified post sinusotomy. In 10, the swelling disappeared eventually at 12 weeks following removal. Two were euthanized due to a lack of response to treatment and the follow up in the other seven unfortunately only had a maximum of 8 weeks, likely unable to identify further complete resolution. In the other post sinusotomy cases where sequestra were not identified and were managed with medical treatment only, decrease or resolution was achieved less consistently and more slowly. 

Woodford and Lane [12] reported the development of two suture exostosis cases in a series of 50 sinusotomies performed by a variety of methods and approaches. Using the 5 cm large trephination access, Quinn et al. [14] reported suture exostosis in a mild form in 36% and marked form with poor cosmetic appearance in 13% of their 60 reported cases. Tremaine and Dixon [11] did report on 277 sinusitis cases of which one out of 115 operated cases were reported with suture exostosis. Fenner et al. [15] reported six cases of suture exostosis in 37 sinusotomies, yet the paper is focused on complications of sinonasal cyst and is likely not a good representation of the true prevalence of suture exostosis. Unfortunately, in the present series, we did not record the number of sinusotomies performed at each collecting institution to allow the establishment of a precise incidence of this complication. Nevertheless, from the present and above-mentioned case series, it can be said that suture exostosis is not a rare surgical complication. From a surgical technique perspective there did not seem to be any sinusotomy or trephination approach prone to the development of suture exostosis in this case series. Two surgeons reported a perceived decrease in the number of cases when shifting to trephinations instead of sinus flaps with an oscillating bone saw (T. Zwick, H. Simhofer; personal observation) but this clinical impression could not be substantiated further and will need more investigation. It should be noted though that the likelihood of creating small sequestration at the corners of a flap is high at locations where the osteotomy line changes direction. This could possibly be overcome by drilling holes at the corners of the bone flap as to have blind ending osteotomy lines that do not cross each other (D. Verwilghen; personal observation). The use of reciprocating saws instead of oscillating saws may negate this issue also. It is likely also important to ascertain the absence of loose bone fragments at the time of closure of the sinusotomy site as to avoid sequester formation in the closure. Further, better surgical planning based on CT images of the head allowing identification of the suture lines could provide sinus access without traumatizing neighboring suture lines.

### 4.3. Idiopathic Cases

Twenty-six cases were presented without any history or signs of either trauma or surgical sinusotomy. Previously, Dixon [1] stated that the apparently high incidence of the condition in thoroughbred horses together with the bilateral symmetry of the swelling would make the trauma theory in these cases unlikely. Yet, considering the various degrees of subtle to intense trauma reported in the traumatic series of cases described above, the identification of sequestra in six idiopathic cases and the histological features of suture lines in unaffected horses this statement may have to be revised. Further, callus formation is reported from the two cases in which histology of idiopathic suture exostosis has been described [3,5]; which would be consistent with the body’s reaction to stabilizing a fracture. 

Although unidentified trauma can never be ruled out and remains the most likely etiological cause of idiopathic suture exostosis cases, other etiopathogenic options should be explored. The growth of the cheek teeth and their relationship with the sinuses, the maxillary and facial bones are a dynamic and evolutive process in the horse. Masticatory forces within this intertwined complex of teeth and bones play a role in the development of facial structures [16]. Compression forces occurring across the naso-frontal and inter-nasal suture during mastication (see Supplementary Video S1) may predispose to a bone reaction in the suture line [17]. Further dental malocclusions may induce excessive forces on suture lines [18] and horses presented with suture exostosis without history and findings of trauma should probably be controlled for the presence of dental malocclusion and or dental eruption issues. Unfortunately, no dental records of the described cases were available for review.

Despite their preference for long bones, in the horse particularly the caudal aspect of the distal radius, osteocartilaginous exostosis—also known as osteochondroma’s—may occur in short bones developing endochondral ossification. Bonilla and Wilson [19] described a case of osteochondroma in the nasal septum concurrent with naso-frontal suture exostosis yet the actual osteochondroma did not appear in the histological analysis of the suture lines. In humans, however, a rare case of fronto-temporo-sphenoidal suture osteochondroma has been described in a 34 year old woman [20] but as yet not in any equids. Histological investigation of more cases may lead to clues in identifying if osteochondromas are involved in the idiopathic expression of this condition. 

A variety of treatments have been initiated in the idiopathic cases. Yet specific outcomes on those cannot be analyzed from the series and further investigations into the management of idiopathic cases should be performed. Two cases in the present series in which the suture exostosis was treated surgically by stabilization of the suture lines as described by Klein and Sacks [5] had a favorable outcome. Both horses became pain-free and the swelling, although still noticeable several months after surgery, notably decreased. The limited time for follow up may not have allowed for the callus to fully remodel. This approach of plate fixation of idiopathic cases has however been debated [2] based on the above mentioned biomechanics of the suture lines and the interactive forces with the stomatognathic apparatus.

### 4.4. Clinical Presentation and Diagnosis

Earlier reports on suture exostosis mention the condition as non-painful [4]. In the current series and independently of the etiopathological classification many horses presented in the acute phase of the condition were reported to be painful at palpation. Treatment should therefore also be focused on providing pain relief, either systemically or locally. 

Whereas the ultimate confirmation of suture exostosis is performed by means of CT, plain radiographs are, however, revealed to be sufficient to establish the diagnosis. Still, X-ray may not be able to identify the presence of sequestra due to difficulties in exposing all specific parts of the suture lines. In the present series, ultrasonography was revealed to be extremely useful in the identification of sequestra and infection pockets in particular cases (Figure 7) and the authors would encourage its use in the workup of suture exostosis cases.

## 5. Limitations and Conclusions

The present series is limited by an inconsistent timeframe for follow up. Therefore, interpretation of the speed of resolution between the different groups should be made with caution. Nevertheless, comparing the different groups, it seems that treated cases (sequestrotomy/management of infection) from the post sinusotomy group were reduced or disappeared more consistently and faster than in the other groups. 

In conclusion, from the present case series it is clear that conservative or medical management of these cases will rarely lead to full clinical resolution of the pathology. Spontaneous disappearance was reported in a number of cases within a 6-month timeframe (except one case where it took 1.5 years) therefore initiating surgical treatment in idiopathic cases could be delayed until after this timeframe. This current case series also demonstrates the importance of the presence of bone sequestra that may be involved in the occurrence of suture exostosis and the need for sequestrectomy in order to speed up recovery. 

## Figures and Tables

**Table 1:** Distribution of 105 equine cases affected with suture exostosis in terms of breed, gender and age.

Breed	*n*	Gender	*n*
American Saddlebred	1	Mare	42
Arabian	1	Gelding	60
Paint	1	Stallion	3
Quarter Horse	5		
Shire	1		
Standardbred	6		
Stock Horse	1		
Thoroughbred	26	AGE in years	
Warmblood	55	Minimum	2
Welsh Cob	6	Maximum	26
Polo Pony	1	Median	10
Connemara	1	Mean	10.46

**Table 2:** Reported season of occurrence and type of housing in 105 equine cases of suture exostosis.

Season	*n*	Housing	*n*
Spring	23	Stabled only	11
Summer	39	Pasture Only	9
Autumn	17	Mixed	71
Winter	17	Unreported	14
Unreported	9		

**Table 3:** History of appearance of suture exostosis in 105 equine cases.

Classification by History	*n*
Swelling appeared following trauma	23
Swelling appeared following surgery	48
Swelling appeared following sinus disease	8
Idiopathic appearance of the swelling	26

**Table 4:** Number of cases reported with epiphora and nasal discharge in 105 equine cases of suture exostosis divided according to history of appearance.

History	Epiphora	Nasal Discharge
Post trauma	9	5
Post surgery	16	24
Post sinus disease	4	5
Idiopathic	10	1

**Table 5:** Method of surgical access to the sinus in 48 cases of equine suture exostosis developed after sinus surgery.

Method of Sinus Access	*n*
Trephination: frontal sinus 5 mm (foley catheter placement)	2
Trephination: 13 mm frontal sinus	9
Trephination: 19 mm frontal sinus	2
Trephination: 24 mm frontal sinus	7
Flap: frontal sinus	10
Flap: maxillary sinus	3
Flap: large fronto-nasal	12
Other	1
Unreported	2

**Table 6:** Number of cases reported with presence of bone sequestra in 105 equine cases of suture exostosis divided according to history of appearance.

History	Sequester
Post trauma	6
Post-surgery	19
Post sinus disease	1
Idiopathic	6

**Table 7:** Follow up information in 88 equine cases with suture exostosis divided according to history of appearance.

History	Cases Available for Follow Up/Total	Outcome
Post trauma	18/23	- In 5 the swelling disappeared after a mean of 25.6 weeks (2 to 36 weeks). - In 4 the swelling was persistent at 26 weeks follow up. - In 9 the swelling had decreased at a mean of 23.5 weeks follow up (4 to 78 weeks).
Post surgery	40/48	- From 19 treated for sequester/infection, o in 10, the swelling disappeared fully after a mean of 6.4 weeks following treatment (SD: 1 to 12 weeks), o in 7, the swelling had reduced at a mean of 5 weeks follow up (SD: 2 to 8 weeks), o one euthanized due to multi-resistant infection and lack of response to treatment after 11 weeks o one euthanized due to persistent infection and development of one sided blepharospasm, increased pain and lack of response to treatment at 7 weeks. - From 5 without further investigation or treatment initiated o in 2, the swelling had slightly decreased at 8 weeks follow up, o in 2, swelling had disappeared after 8 weeks, o in 1, horse was seen 2 years after without swelling—duration before disappearance unknown. - From 16 in which medical treatment was initiated (local and systemic NSAIDS (phenylbutazone, flunixine systemically, diclofenac topical)/Antimicrobials), o in 6, swelling disappeared within mean 5.4 weeks (SD: 4 to 6 weeks), o in 10, swelling decreased at a mean follow up of 10.4 weeks follow up (4 to 24 weeks).
Post sinus disease	5/7	- Following mass/cyst removal, o in 2, swelling had disappeared at 6 months follow up, o in 2, swelling had slightly decreased at 6 months follow up, o in 1, swelling unchanged at 1 year follow up.
Idiopathic	25/26	- In 5, swelling disappeared after mean of 25 weeks (4 to 78 weeks). - In 1, at follow up 10 years later swelling had disappeared yet timeframe for disappearance was unknown. - In 17, swelling decreased but persisted after a mean follow up of 48.6 weeks (8 to 208 weeks). - In 2, with internal fixation slight decrease was observed at, respectively, 8 and 9 months follow up.

## Data Availability

Collated anonymized patient data available on request.

## Supplementary Materials

The following supporting information can be downloaded at: https://www.mdpi.com/article/10.3390/vetsci9070365/s1, Video S1: Horse with suture line separation illustrating the movement caused in the suture line during mastication (courtesy Chris Pearce).
